# Rapid and Slow Progressors Show Increased IL-6 and IL-10 Levels in the Pre-AIDS Stage of HIV Infection

**DOI:** 10.1371/journal.pone.0156163

**Published:** 2016-05-23

**Authors:** Rúbia M. de Medeiros, Jacqueline M. Valverde-Villegas, Dennis M. Junqueira, Tiago Gräf, Juliana D. Lindenau, Marineide G. de Mello, Priscila Vianna, Sabrina E. M. Almeida, Jose Artur B. Chies

**Affiliations:** 1 Technological and Scientific Development Center - CDCT, State Foundation in Production and Health Research - FEPPS, Rio Grande do Sul, Porto Alegre, Brazil; 2 Post Graduation Program in Genetic and Molecular Biology, Federal University of Rio Grande do Sul, Porto Alegre, Brazil; 3 Uniritter Laureate International Universities, Health Science Department, Porto Alegre, Brazil; 4 Post Graduation Program in Biotechnology and Biosciences, Federal University of Santa Catarina, Florianópolis, Brazil; 5 Infectious Disease Service, Nossa Senhora da Conceição Hospital, Porto Alegre, Brazil; University of Missouri-Kansas City, UNITED STATES

## Abstract

Cytokines are intrinsically related to disease progression in HIV infection. We evaluated the plasma levels of Th1/Th2/Th17 cytokines in extreme progressors, including slow (SPs) and rapid (RPs) progressors, who were thus classified based on clinical and laboratory follow-up covering a period of time before the initiation of HAART, ranging from 93–136.5 months for SPs and 7.5–16.5 months for RPs. Analyses were also performed based on the different stages of HIV infection (chronic, pre-HAART individuals—subjects sampled before initiating HAART but who initiated therapy from 12 to 24 months—and those receiving HAART). The plasma cytokine levels of 16 HIV-infected rapid progressors and 25 slow progressors were measured using a Human Th1/Th2/Th17 CBA kit. The IL-6 and IL-10 plasma levels differed significantly between the stages of HIV infection. The IL-6 levels were higher in slow progressors pre-HAART than in chronically infected SPs and HIV-seronegative individuals. The IL-10 levels were higher in slow progressors pre-HAART than in slow progressors receiving HAART and HIV-seronegative controls, and in rapid progressors, the IL-10 levels were higher in pre-HAART subjects than in HIV-seronegative controls. The results reflect the changes in the cytokine profile occurring during different clinical stages in HIV+ subjects. Our results suggest an association between increased IL-6 and IL-10 levels and pre-HAART stages independent of the slow or rapid progression status of the subjects. Thus, increased IL-6 and IL-10 levels could indicate a global inflammatory status and could be used as markers of the disease course in HIV-infected individuals.

## Introduction

HIV infection progression is commonly defined based on the stability of CD4+ T-cell counts, viral load and the duration of symptom-free HIV infection [1,2]. Subjects with stable CD4+ T-cell counts and other clinical and immunological parameters over a period ranging from 7 to 10 years or more are known as ‘slow progressors’ (SPs). These individuals represent between 5% and 15% of the HIV-infected population [3,4]. Conversely, approximately 5% of the HIV-infected subjects progress to AIDS within 3 years after viral infection and, based on this time progression, are termed ‘rapid progressors’ (RPs) [3,5].

Plasma cytokine levels have been postulated to change dramatically over the course of HIV infection [6–8]. This variation involves a change from an environment characterized predominantly by T-helper type 1 cytokines, associated with cell-mediated immune responses, to an environment in which T-helper type 2 cytokines, known to enhance humoral immune responses, are dominant [9–11]. In recent times, however, *cytokinology* has evolved, and multiple T helper populations, such as Th17, and a number of different cytokine functions have been identified and analyzed in the context of HIV infection [12–14]. Although several studies suggest that cytokine levels are different in distinct stages of HIV infection, little is known about this topic in subjects classified according to rapid or slow disease progression.

The understanding of the cytokine profile throughout the course of HIV infection will contribute to the elucidation of the relationships between the immune response and the HIV infection outcome, ultimately improving clinical monitoring. Roberts *et a*l. (2010) and Liovat *et al*. (2012) observed a relationship between an increase in plasma viral load, a decline in CD4+ T-cell counts and an increase of certain cytokine levels in HIV-infected subjects and suggested the predictive value of these cytokines for disease progression [15,16]. In the present study, we evaluated the Th1/Th2/Th17 cytokine plasma levels in both the extreme progressor groups (SPs and RPs—thus classified taking into account clinical and laboratory follow-up covering a period of time before the initiation of HAART ranging from 93–136.5 months for SPs and 7.5–16.5 months for RPs). The cytokine evaluation also took into account the different stage of HIV infection in each HIV-seropositive subject. Our results suggest an association between increased IL-6 and IL-10 levels and stages of infection pre-HAART, independent of the slow or rapid progression status of the patient. Thus, increased IL-6 and IL-10 levels could indicate a global inflammatory state and could be used as markers of disease progression in HIV-infected subjects.

## Methods

### Enrollment of the study population

We reviewed >3,500 medical records of HIV-infected individuals regularly attended in the Infectious Disease Service at Nossa Senhora da Conceição Hospital, Porto Alegre city between 2011 and 2013 to select rapid and slow AIDS progressors. To estimate their AIDS progression profiles (described below), longitudinal clinical and laboratory data were used, including CD4+ T-cell counts, plasma viral loads, stage of HIV infection at the time of sample collection and highly active antiretroviral therapy (HAART) prescriptions. In addition, their demographic data were obtained (Table 1). This study received the ethical approval of the Nossa Senhora da Conceição Hospital Ethical Committee (Project Number 10–123).

**Table 1:** Clinical baselines and demographic characteristics of the 41 HIV-infected subjects enrolled in this study as rapid or slow disease progressors.

	No. of subjects	
	RP (n16)	SP (n25)
**Baselinemeasurements**		
**Time progression**^a^	01 (1–3)	11 (10–13)
**First CD4+ T cell count**^b^	487 (356–600)	553 (392–712)
**First RNA viral load**^c^	3.32 (1.84–4.25)	2.2 (1.79–3.96)
**Follow-up duration**^d^^,^^e^	13.5 (7.5–16.5)	111 (93–136.5)
**Slope CD4+ T-cell count** ^e^	-0.66 (-1.28, -0.27)	0.40 (0.22, 0.54)
**Median RNA viral load**^e^	4.28 (3.8–4.79)	3.73 (3.23–4.10)
**Demographiccharacteristics**		
**Median age** ^a^	38 (32–50)	42 (35–48)
**Sex**		
Female	12 (0.75)	22 (0.88)
Male	4 (0.25)	3 (0.12)

RP rapid progressor; SP slow progressor

^a^median (IQ), years;

^b^median (IQ), cells/mm^3^;

^c^median (IQ), log_10_ copies/mL;

^d^median (IQ), months;

^e^estimated for data pre-HAART.

### Characterization and stage of HIV-infected progressors

**Fig 1:**
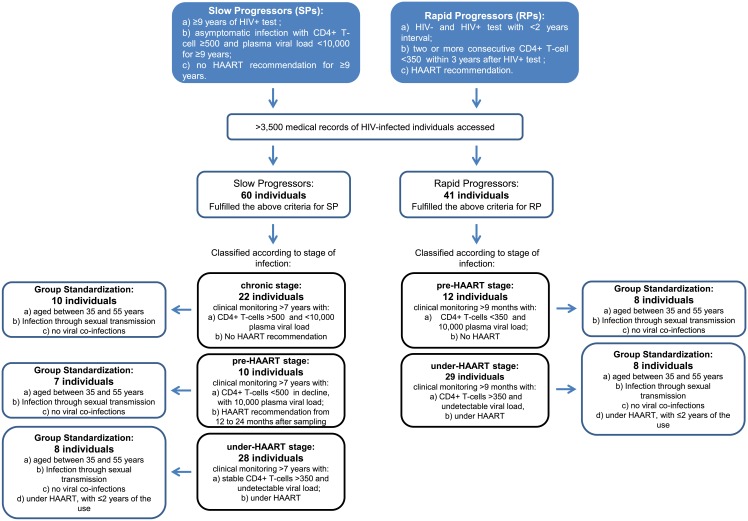
Flowchart of the sampling procedure.

Based on the medical records data, the HIV-infected subjects were retrospectively classified into two groups: 16 RPs and 25 SPs (Fig 1). For RPs, the time of HIV seroconversion was estimated as the midpoint between the times of the last documented HIV-seronegative test and the first HIV-seropositive test within a maximum interval of 2 years. RPs were defined as subjects who had two or more CD4+ T-cell measurements <350 cells/mm^3^ within 3 years of seroconversion and were recommended to initiate HAART. SPs were defined as subjects with asymptomatic HIV infection ≥9 years after diagnosis, with average CD4+ T-cell measurements of ≥500 cells/mm^3^ and plasma viral loads<10,000 copies/mL throughout the years. The median follow-up time before the initiation of HAART was 111 months (93–136.5 months, interquartile range) for SPs and 13.5 months (7.5–16.5 months, interquartile range) for RPs. All longitudinal retrospective data were regularized and normalized for statistical analyses, as described in S1 Text.

Samples collected from the RPs and SPswere also classified according to stage of infection (Fig 1). For SPs: 10 subjects were in the chronic stage (stable CD4+ T-cells >500 cells/mm^3^ with <10,000 copies/mL plasma viral load), 7 subjects were in the pre-HAART stage [CD4+ T-cells <500 cells/mm^3^ in decline, with 10,000 copies/mL plasma viral load; the subjects were sampled before initiating HAART, but they initiated therapy from 12 to 24 months (median 16 months) after sampling] and 8 subjects were in the under-HAART stage (stable CD4+ T-cells >350 cells/mm^3^ and undetectable plasma viral loads; the subjects were sampled while under HAART). For the RPs: 8 subjects were in the pre-HAART stage (CD4+ T-cells <350 cells/mm^3^, with 10,000 copies/mL plasma viral load; the subjects were sampled before initiating HAART) and 8 subjects were in the under-HAART stage (stable CD4+ T-cells >350 cells/mm^3^ and undetectable plasma viral loads; the subjects were sampled while under HAART).

In addition, samples from eight HIV-seronegative voluntary donors, with no known metabolic disorders or other medical conditions at the time of blood collection, were used as controls. The HIV-seronegative samples were matched on the basis of sex and ethnic origin.

### Th1/Th2/Th17 cytokine profile

Approximately 8 mL blood was collected from each participant after they signed a consent form. The blood was centrifuged, and the plasma was stored at −80°C. Cytokine analysis was performed using the Human Th1/Th2/Th17 Cytometric Bead Array kit (CBA; BD Biosciences, San Jose, CA, USA; Catalog No. 560484), which allowed the simultaneous detection of IL-2, IL-4, IL-6, IL-10, TNF-α, IFN-γ and IL-17A. Aliquots of plasma were diluted with assay diluent (1:2 v/v), and CBA analysis was performed as per the manufacturer’s instructions. Two hundred microliters of each sample was plated on PRO-BIND^™^ 96-well assay plates and analyzed on the FACS Array Bioanalyzer using FCAP FCS Filter and FCAP Array Software (BD Biosciences). Using these software packages, the debris were filtered from the data, and identification of the bead populations and their mean fluorescence intensities (MFIs) was performed.

### Statistical analysis

Global comparisons of the circulating cytokine levels (adjusted by log transformation) were performed for 49 selected individuals using the Kruskal–Wallis test. Two-by-two group comparisons were performed on the basis of Mann–Whitney U tests, and correlations between cytokine levels and clinical data were calculated using Spearman coefficients. The false discovery rate (FDR) procedure described by Benjamini and Hochberg (1995) was used to account for multiple comparisons.

## Results

### Description of study participants

Clinical data from infection, specifically the CD4+ T-cell counts and plasma viral loads, were consistent with those previously described for RPs and SPs (Table 1). RPs showed higher plasma viral loads and lower CD4+T-cell counts (p < 0.001) than SPs at the first time point. Furthermore, the CD4+ T-cell counts decreased significantly (p < 0.001) faster for RPs (−0.66 CD4+ T-cell slope; −1.28, −0.27 IQ) than for SPs (0.40 CD4+ T-cell slope; 0.22, 0.54 IQ). SPs and RPs did not differ significantly in age, sex or ethnicity. No relationships between the exposure category and progression groups were observed, and chronic viral co-infections, such as HCV, HBV and HTLV, diagnosed in the first year of follow-up, did not differ between the extreme phenotypes (data not shown).

### Cytokine profiles in rapid or slow progressors in different stages of HIV infection

**Fig 2:**
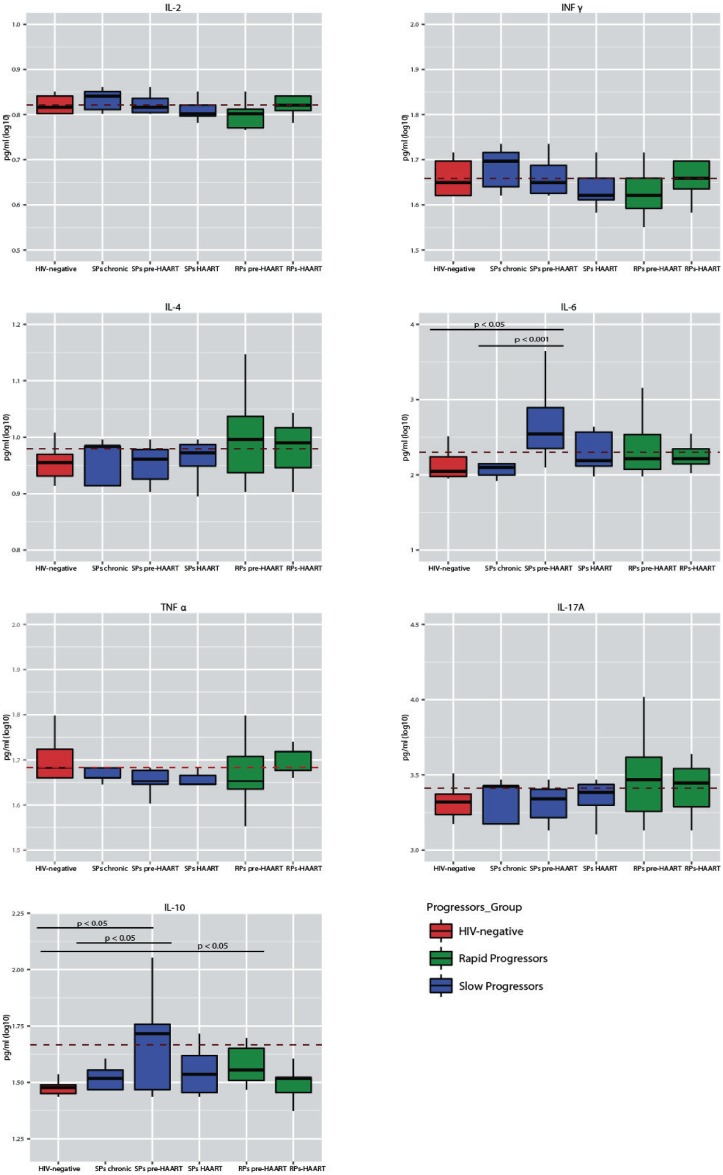
Plasma cytokine levels in rapid and slow progressors subgrouped according to clinical stage. Plasma levels of the cytokines IL-2, IFN-γ, IL-4, IL-6, TNF-α, IL-17A and IL-10 in rapid (green) and slow (blue) progressors grouped by different stages of HIV infection and in HIV-seronegative controls (red). A Kruskal Wallis test showed significant differences between the IL-6 levels for chronically infected*vs* pre-HAART SPs (p = 0.001) and pre-HAART SPs*vs* HIV-seronegative individuals (p = 0.01). In addition, significant differences were observed in the IL-10 levels between pre-HAART SPs*vs* SPs receiving HAART (p = 0.04); pre-HAART SPs*vs* HIV-seronegative individuals (p = 0.02); and pre-HAART RPs*vs* HIV-seronegative individuals (p = 0.01). After FDR, significant differences were maintained for the IL-6 levels in chronically infected*vs* pre-HAART SPs (p = 0.03) and the IL-10 levels in pre-HAART RPs*vs* HIV-seronegative individuals (p = 0.01).

**Fig 3:**
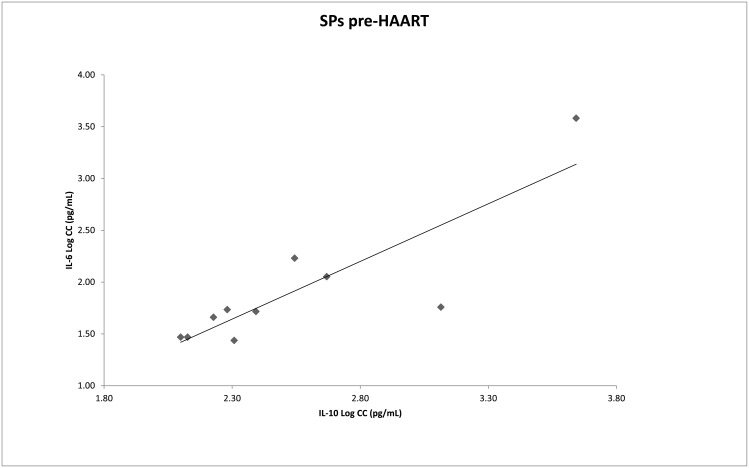
IL-6 and IL-10 plasma levels in slow progressors in a pre-HAART stage. A significant Spearman correlation, rho = 0.750, was observed between theIL-6 and IL-10 plasma levels in pre-HAART SPs (p = 0.02).

We assessed the circulating IL-2, IL-4, IL-6, IL-10, IL-17A, TNF-α and IFN-γ levels in RPs and SPs in different stages of HIV infection and in HIV-seronegative control individuals (Fig 2 and Table 2). Global comparisons revealed that the IL-6 and IL-10 levels differed between the stages of HIV infection (p = 0.03 and p = 0.02, respectively). Two-by-two evaluations were performed within each group classified according to the stages of HIV infection. Among the SPs, the IL-6 levels were higher in the pre-HAART stage (2.59 pg/mL) than in the chronic stage (2.15 pg/mL, p = 0.001), and the IL-10 levels were higher in those in the pre-HAART stage (2.18 pg/mL) than in patients receiving HAART (1.55 pg/mL, p = 0.04). Moreover, the IL-6 (2.59 pg/mL) and IL-10 (2.18 pg/mL) levels of the SPs in the pre-HAART stage were significantly higher than the IL-6 (2.13 pg/mL, p = 0.01) and IL-10 (1.47 pg/mL, p = 0.02) levels in HIV-seronegative controls. In addition, the IL-10 levels were significantly higher in pre-HAART RPs(1.74 pg/mL) than in HIV-seronegative controls (1.47 pg/mL, p = 0.01). After the application of the FDR procedure, significant differences revealed higher IL-6 levels in chronically infectedSPs than in pre-HAART SPs (p = 0.03) and higher IL-10 levels in pre-HAART RPs than in HIV-seronegative controls (p = 0.01). No statistically significant differences were observed between the RP and SP groups. The investigation of the correlation between cytokine levels, the slope of change in CD4+ T-cell numbers and the viral load revealed that an increase in IL-6 levels correlated with an increase in the plasma viral load in SPs with>500 CD4+ T-cells (rho = 0.744, p = 0.014). With respect to pre-HAART SPs, a positive correlation between the IL-6 and IL-10 levels was observed (rho = 0.750, p = 0.02) (Fig 3). Furthermore, in pre-HAART SPs, a correlation between an increase in the TNF-α levels and a decrease in the CD4+ T-cell slope (rho = −0.686, p = 0.04) was observed. In pre-HAART RPs, an increase in the TNF-α levelswas correlated with an increase in the viral load (rho = 0.747, p = 0.033).

**Table 2:** IL-2, IL-4, IL-6, IL-10, TNF-α, IFN-γ and IL-17A plasma levels in HIV-seronegative control and HIV-seropositive individuals grouped by different clinical stages of HIV infection.

Group and clinic stage (n)	Female sex	Median age	CD4+ T-cell sampling	VL log sampling	IL2		IL4		IL6		IL10		TNF		INF		IL17	
**HIV-seronegative (8)**	6	35	---	---	0.82	(0.02)	0.95	(0.03)	**2.13**^c^	(0.19)	**1.47**^d^^,^^e^	(0.03)	1.70	(0.05)	1.66	(0.04)	3.32	(0.12)
**SPs chronic (10)**	9	40	802 (560–1047)	3.49 (2.0–3.9)	0.83	(0.03)	0.96	(0.04)	**2.15**^a^	(0.28)	1.51	(0.05)	1.67	(0.07)	1.68	(0.05)	3.34	(0.13)
**SPs pre-HAART (7)**	6	41	496 (375–630)	4.25 (3.6–4.5)	0.85	(0.10)	1.01	(0.16)	**2.59**^a^^,^^c^	(0.53)	**2.18**^b^^,^^d^	(1.12)	1.72	(1.12)	1.71	(0.20)	3.53	(0.59)
**SPs HAART (8)**	7	42	601 (370–936)	1.69	0.81	(0.04)	0.96	(0.03)	2.30	(0.27)	**1.55**^b^	(0.11)	1.65	(0.05)	1.65	(0.07)	3.35	(0.12)
**RPs pre-HAART (8)**	6	36	316 (197–387)	4.09 (3.8–4.5)	0.79	(0.05)	1.00	(0.08)	2.35	(0.39)	**1.74**^e^	(0.37)	1.67	(0.08)	1.60	(0.09)	3.48	(0.28)
**RPs HAART (8)**	6	35	730 (512–858)	1.69	0.82	(0.02)	0.99	(0.07)	2.26	(0.17)	1.50	(0.07)	1.69	(0.05)	1.66	(0.04)	3.46	(0.26)

Mean (Std. Error).

Kruskal Wallis test:

^a^ SPs chronic vs SPs pre-HAART (p = 0.001);

^b^ SPs pre-HAART vs SPs under-HAART (p = 0.04);

^c^ SPs pre-HAART vs HIV-seronegative (p = 0.01);

^d^ SPs pre-HAART vs HIV-seronegative (p = 0.02);

^e^ RPs pre-HAART vs HIV-seronegative (p = 0.01).

After FDR, significant differences were IL-6 levels in SP chronic vs SP pre-HAART (p = 0.03) and IL-10 levels in RP pre-HAART vs HIV-seronegative (p = 0.01).

## Discussion

Three decades have elapsed since researchers began to understand HIV infection pathways. Considering the disease course, from the exposure to the virus, through the different clinical symptoms, up to AIDS development, it is now possible to identify several outcomes [1,4,5]. However, the identification of the biological factors modulating the immune system and, consequently, the immune response that potentially segregates the extreme groups of AIDS progressors (namely, SPs and RPs) remains a challenge.

Our experimental design aimed to quantify the actual Th1/Th2/Th17 cytokine plasma levels in HIV+ individuals. Although at first sight, the sample size seems to be reduced, it was obtained through a methodological approach that ensured the homogeneity of the samples and therefore allowed us to have a high degree of confidence in the results (Fig 1). Stringent sampling was mandatory to avoid the possibility that variable factors masked the effects of the clinical stage of infection. As a result of the stringent inclusion criteria, the sample groups used in this study are homogeneous in size, age, gender, co-infection presence and treatment time. The sample selection method reduced potential bias, focusing on specific groups. In this sense, it was possible to evaluate the cytokine profiles of individuals with extreme rates of progression (i.e., slow and rapid progressors) in well-characterized clinical stages (i.e., in a chronic period and immediately before AIDS development).

Despite the extreme difference in the duration of symptom-free HIV infection, the SP and RP groups presented increased levels of IL-6 and IL-10 during the pre-HAART stage, suggesting a similar pattern in the immunological response in both groups. While the SP subjects in the chronic infection stage maintained relatively constant IL-6 and IL-10 levels, the SP pre-HAART group (composed of subjects who had CD4+ T-cell counts >500 cells/mm^3^ at the first collection and then underwent a decrease in CD4+ T-cell counts to <350 cells/mm^3^ within 24 months from sampling) revealed a significant increase in IL-6 levels over time. The same trend was observed for the IL-10 levels, although, probably due to a higher variance, the differences did not reach statistical significance. In addition, the IL-6 and IL-10 levels were higher in pre-HAART SPs than in HIV-seronegative controls.

An increase in IL-6 levels has already been associated with the development of opportunistic diseases and mortality in HIV infection [17]. Recently, Williams et al. (2013) showed a significant increase in the plasma IL-6 levels in HIV-seropositive subjects (average of 323.46 CD4+ T-cells/mm^3^) compared with HIV-seronegative subjects, demonstrating the important role of IL-6 in the course of the infection [18]. Along the same lines, different studies reported that the transfection of a human astrocyte cell line with a plasmid encoding any of several different HIV proteins (gp120, Nef, Tat or Vpr) resulted in the increased expression of IL-6 through the activation of transcription factors such as NF-κB[19–22]. Therefore, it could be suggested that the increased availability of HIV particles in the pre-HAART stage is involved in the observed increased in the IL-6 level. With respect to IL-10, increased levels of this cytokine are observed with disease progression in chronically HIV-infected subjects, and decreased levels are observed with HAART initiation [23]. Additionally, Roberts et al. (2010) showed that significantly elevated IL-10 levels during acute HIV infection were directly associated with the acute infection viral loads and a high risk of CD4+ T-cell loss [15]. Taken together with our observations, these results suggest a strong association of high IL-6 and IL-10 levels with progression through the stages of HIV infection to AIDS regardless of the patients’ progression group.

In addition, our study evaluated the relationship among the classical markers of AIDS progression, i.e., CD4+ T-cell count, median viral load and cytokine levels, and revealed a correlation between increased viral load and increased IL-6 levels (rho = 0.75, p = 0.02). Moreover, in pre-HAART SPs, the IL-6 and IL-10 levels were positively correlated (rho = 0.75, p = 0.01) (Fig 3). The pro-inflammatory role of IL-6 (mononuclear cell recruitment, inhibition of T-cell apoptosis, inhibition of T_reg_ cell differentiation and activation of Th17 cell differentiation) has been widely discussed (for a review, see Rose-John, 2012) [24]. Conversely, it has been observed that the expression of IL-10, an anti-inflammatory cytokine, is induced at a later stage of acute HIV infection, after the first burst of pro-inflammatory cytokines [23,25,26]. Liovat et al. (2012) observed that rapid progressors (defined by the accelerated loss of CD4+ T-cells in a follow-up 42 months post-seroconversion) had higher levels of cytokines during acute HIV infection than typical or slow progressors [16]. These authors also suggested that IL-10 is induced as a consequence of the strong inflammatory response, in an attempt to inhibit the exacerbation of immune responses. Therefore, we may speculate that the viral load and IL-6 levels concomitantly increase in the SP group, leading to an increase in the IL-10 levels. However, IL-10 plays a potent anti-inflammatory role, and therefore, it could inadvertently promote viral persistence through the inactivation of effective immune responses. Although it is not possible to conclusively identify increased levels of IL-6 and IL-10 as the direct causes or consequences of progression to AIDS, our results strongly suggest that modifications in the cytokine profile could be used as markers of a global inflammatory state and, consequently, of the disease course, even in HIV infections with extreme phenotype progression.

In the RP group, we observed lower levels of IL-2 and IFN-γ and higher levels of IL-4, IL-6 and IL-17A compared to the SPs or the HIV-seronegative controls, although these differences were not statistically significant. These observations suggest that there is a predominant Th2 (IL-4, IL-6) and Th17 (IL-17A) response during early/acute HIV infection in RPs. To this point, both T cell subsets (Th2 and Th17) were described as susceptible/permissive to HIV replication and are reduced in chronic stages of HIV infection [13,27–29]. Some authors have suggested that Th17 cells have a dual impact on HIV infection. In the acute phase of infection, Th17 cells could promote cell migration to the gut and favor viral replication. Several reports have discussed the Th1-to-Th2 shift in AIDS, and it can be suggested that an increased Th17 response and a possible favoring of HIV replication in the early stages of infection in RPs would accelerate disease progression to AIDS [10,30,31].

In conclusion, slow and rapid progressors show elevated IL-6 and IL-10 levels in the pre-HAART stage. Furthermore, although the relationship between IL-6 and IL-10 is unclear in HIV+ subjects, these cytokines appear to be intrinsically linked to infection progression and AIDS onset. Thus, they could be of great value in the clinical follow-up of HIV+ individuals. On the basis of our findings, we suggest that IL-6 and IL-10 measurement should be incorporated into the clinical management of HIV-infected subjects as a valuable tool in both the decision of HAART initiation and the surveillance of immune activation and inflammation. Finally, the predominant Th2 and Th17 profiles in early HIV infection observed in rapid progressors should be better investigated because if they are corroborated by other studies, this phenotype will define an immune profile in early stages of infection, which will be crucial for decision-making in therapy.

## Supporting Information

S1 Text Regularization and normalization of the longitudinal retrospective clinical data. (DOC)
