# Supplementary material for: Rapid and Slow Progressors Show Increased IL-6 and IL-10 Levels in the Pre-AIDS Stage of HIV Infection
Source: PLoS One. 2016 May 23;11(5):e0156163. doi: 10.1371/journal.pone.0156163 (PMC4877004; doi:10.1371/journal.pone.0156163)
Supplement: S1 Text — (DOC) [file pone.0156163.s001.doc]

**Supporting Information**

**S1Text Regularization and normalization of the longitudinal retrospective clinical data**

The clinical follow-up of HIV-infected patients involves dynamic measures that change during the time to AIDS progression. The current study retrospectively collected data, for both groups in 85% the observing a mean interval among the measurements of 6 months for SP and 3 months for RP. Thus, all measurements were regularized by weighted average of all collections done in this time intervals to calculate CD4+ T cells slope and viral load median. The viral load values are presented in log transformation, and CD4 values were normalized. The median follow-up time before the initiation of HAART was 111 months (93-136.5 months, interquartile range) for SP and 13.5 months (7.5-16.5 months, interquartile range) for RP.
